# Interaction of genotype and diet on small intestine microbiota of Japanese quail fed a cholesterol enriched diet

**DOI:** 10.1038/s41598-018-20508-9

**Published:** 2018-02-05

**Authors:** Shasha Liu, Hein Min Tun, Frederick C. Leung, Darin C. Bennett, Hongfu Zhang, Kimberly M. Cheng

**Affiliations:** 1 The State Key Laboratory of Animal Nutrition, Institute of Animal Sciences, Chinese Academy of Agricultural Sciences, Beijing, China; 2 Avian Research Centre, Faculty of Land and Food Systems, University of British Columbia, Vancouver, British Columbia Canada; 3 School of Biological Sciences, Faculty of Science, University of Hong Kong, Hong Kong SAR, China; 4 Department of Pediatrics, University of Alberta, Alberta, Canada; 5 Animal Science Department, California Polytechnic State University, San Luis Obispo, California, USA

## Abstract

Our previous study has shown that genetic selection for susceptibility/resistance to diet-induced atherosclerosis has affected the Japanese quail’s cecal environment to accommodate distinctly different cecal microbiota. In this study, we fed the Atherosclerosis-resistant (RES) and -susceptable (SUS) quail a regular and a cholesterol enriched diet to examine the interaction of host genotype and diet on the diversity, composition, and metabolic functions of the duodenal and ileal microbiota with relations to atherosclerosis development. In the duodenal content, 9 OTUs (operational taxonomic units) were identified whose abundance had significant positive correlations with plasma total cholesterol, LDL level and/or LDL/HDL ratio. In the ileal content, 7 OTUs have significant correlation with plasma HDL. Cholesterol fed RES hosted significantly less *Escherichia* and unclassified *Enterobacteriaceae* (possibly pathogenic) in their duodenum than SUS fed the same diet. Dietary cholesterol significantly decreased the duodenal microbiome of SUS’s biosynthesis of Ubiquinone and other terpenoid-quinone. Cholesterol fed RES had significantly more microbiome genes for Vitamin B6, selenocompound, taurine and hypotaurine, and Linoleic acid metabolism; Bisphenol degradation; primary bile acid, and butirosin and neomycin biosynthesis than SUS on the same diet. Microbiome in the ileum and ceca of RES contributed significantly towards the resistance to diet induced atherosclerosis.

## Introduction

Atherosclerosis is one of the leading causes of mortality in developed countries, and with increasing incidents in the developing countries^1^. It is a complex disease affected by the interaction of genetic and environmental risk factors^2–4^. In the past decade, research efforts in the biomedical field have focused on the implications of gut microbiome on human diseases. There is evidence that symptomatic atherosclerosis is associated with an altered gut microbial community in human and mice^5–7^. It has been demonstrated that cecal microbial transplantation from a susceptible strain to wild-type mice enhanced choline diet-dependent atheroslceorosis and TMAO (trimethylamine-*N*-oxide) levels in wild-type mice^8^. Examination of human oral and atherosclerotic plaque microbiota in patients with clinical atherosclerosis suggests that microbes from the oral cavity and perhaps the gut may be a reservoir for bacteria found in atherosclerotic plaques^9^. Our previous study has also shown that genetic selection for susceptibility/resistance to diet-induced atherosclerosis has affected the Japanese quail’s cecal environment to accommodate distinctly different cecal microbiota^10^. Most published studies have compared the fecal or cecal micriobial communities between atherosclerosis and healthy subjects, but there has been sparse information on the comparison of cecal and small intestinal microbiota in the same subject.

Mapping of bacterial communities in human, mouse and chicken intestinal tract^11–14^ revealed variations of microflora composition along the gastrointestinal tract relating to factors such as nutrient availability, pH and oxygen concentrations^15^. The small intestinal microbiota have the capacity to affect the absorption and metabolism of nutrients, the immune system, and defending the host from invasion of pathogenic bacteria^16–18^. While there have been studies on the intestinal microbiota metabolism of L-carnitine to TMAO^19,20^, the diversity and composition of microbes in the small intestine under healthy and disease states is still an area of ongoing research.

The atherosclerosis-resistant (RES) and -susceptible (SUS) Japanese quail^21^ is a proven animal model for studying atherosclerosis^22,23^. The two strains were developed by divergent selection from a common foundation population^21^. When challenged with a high cholesterol diet, about 80% of the SUS males will develop atherosclerosis whereas only about 4% of the RES males will. They host cecal microbial communities similar to that in mice and human^24^. In the current study, we fed the RES and SUS quail (from here on referred to as RES and SUS) a regular (control) and a cholesterol enriched diet, to examine the interaction of host genotype and diet on the diversity and composition of duodenal and ileal microbiota with relations to atherosclerosis development. We further included data from our cecal microbiota study^24^ for comparison and functional predictions.

## Methods

### Experimental Design

Duodenum and ileum contents were collected from the same individuals of RES and SUS whose cecal contents were analyzed in Liu *et al*.^24^. The experimental protocol has been described in Liu *et al*.^24^. Briefly, 80 RES and 80 SUS males were fed a semi-synthetic diet prepared according to the National Research Council (NRC) nutrient requirements standards recommended for Japanese quail (http://www.nap.edu/catalog/2114.html) from hatching to 6 weeks of age. At six weeks of age, they were divided into two dietary treatment groups and fed either the semi-synthetic diet (control) or the semi-synthetic diet with additional cholesterol (0.5% w/w) for another 6 weeks^22^. Individual birds were identified by numbered wing bands. Both RES and SUS fed the same diet were kept in the same pen. Birds on the alternative diet were kept in a neighbouring pen. The two side-by-side pens should have similar microbiological environment. At 12 weeks of age, six birds from each of the four treatment groups with body weight closest to the mean of the population were euthanized by decapitation and trunk blood was collected into Vacutainer tubes containing lithium heparin, and centrifuged at 4 °C for 10 min at 3,000 × g. Plasma was stored at −20 °C until it was later used for lipid analysis^24^. The aortic tree (the brachycephalic arteries to their bifurcations and the aorta to the iliac branching) of each bird was dissected out, opened longitudinally and examined under a 10–30X dissecting microscope for a semi-quantitative scoring^23^ of the seriousness of the atherosclerotic lesions on the interior wall. A score of 0 (normal) to 4 (presence of severe atherosclerotic lesions) was assigned by two independent scorers who were blind to the genetic and diet status of the bird. Segments of duodenum (The U-shape section at the beginning of small intestine) ileum (section between Jejunum and the large intestine), and ceca, including gut content were collected from each bird. All samples were quick frozen on dry ice immediately after collection and stored at −70 °C until processed for DNA extraction. The cecal microbiome was examined and reported in Liu *et al*.^24^.

### DNA Extraction and Pyrosequencing

Dissected duodenum and ileum segments were thawed and the contents were gently scraped into sterile vials. Genomic DNA was extracted from samples (0.2–0.5g/sample) using the PowerMax Soil DNA Isolation Kit (MO BIO laboratories. Inc., Carlsbad, CA) according to the manufacture’s instructions. PCR amplifications were performed using the FastStart high fidelity PCR system (Roche Molecular Diagnostics, Branchburg, NJ, USA). The variable region 3–5(V3–V5) of the bacterial 16S rRNA gene was amplified with a primer set of 341F (5′-ACT CCT ACG GGA GGC AGC AG-3′) and 926R (5′-CCG TCA ATT CMT TTG AGT TT-3′) with the sample specific forward primer bearing a multiplex identifier (MID) sequences. All 341F and 926R primers were modified with adaptor A and B sequences respectively for pyrotag sequencing.The amplification program consisted of an initial denaturation step at 94 °C for 2 min; 32 cycles of denaturation at 94 °C for 30 s, annealing at 58 °C for 30 s, and elongation at 72 °C for 30 s; and a final extension step at 72 °C for 7 min. The size of the PCR products was confirmed by gel electrophoresis.The PCR products was then purified using Gel extraction kit (Invitrogen) and were quantified using the NanoDrop 2000 (Thermo Scientific, Wilmington, DE, USA). The Amplicon libraries were subjected to pyrotag sequencing using a bench-top 454 GS Junior (454 Life Sciencesa Roche Company, Branford, CT, USA) with the GS Junior Titanium Sequencing Kit (https://lifescience.roche.com/shop/en/us/products/gs-junior-titanium-sequencing-kit).

### Sequence Analysis

The raw 16s rRNA sequences were processed using Quantitative Insights Into Microbial Ecology (QIIME) software package^25^. Raw sequences were filtered to meet the following quality criteria: (1) complete barcode sequences followed by a forward primer sequence, with no mismatch in either barcode or primer sequence; (2) reads lengths between 150 and 900 bases; (3) average quality score >25; and (4) homophlymer run of 8 nt. De-noising of the dataset was performed using DENOISER v. 0.9.1^26^ as implimented in QIIME. Chimeric sequences were removed using Chimera Slayer (http://microbiomeutil.sourceforge.net/). The filtered sequences were assigned to groups basing on their respective barcode sequences. Similar sequences were assigned into operational taxonomic units (OTUs) at a pairwise identify of 97% using UCLUST (http://www.drive5.com/usearch/). Representative sequence was the most abundant sequence in each OTU. Representative sequences (at 97% similarity) were then classified taxonomically using Ribosomal Database Project (RDP) classifier 2.0.1^27^. The OTUs were aligned using PyNAST with a minimum alignment length of 150 bp and a minimum percent identity of 75%^28^. After alignment, PH LANE mask (http://greengenes.lbl.gov/) was conducted to screen out the hypervariable regions.

### Statistical Analysis

#### Richness and Diversity Indices

Rarefaction Plots were constructed and Alpha-diversity (Chao1 Richness, Simpson’s and Shannon’s Diversity indices) were estimated as implemented in QIIME^29^. Beta-diversity among microbial communities was compared using Principal Component Analysis (PCA) in SIMCA-P (version 14.0) to visualize microbial community’s patterns caused by diet and host genotype. Results of the PCA were statistically tested by multivariate analysis of variance (MANOVA)^30^.

#### Comparison of Microbial Communities

Bacterial abundance difference at the genus level and plasma lipid parameters were examined by multivariate analysis, using Tukey’s HSD/ANOVA for mean separation (SPSS 13.0; SPSS Institute, 2001). *P* < 0.05 was considered significant,

Venn diagrams^31^ and “nearest-shrunken centroid” (NSC) classification^32^ were used to detect core microbiota community which best characterize each group. From each treatment group, the OTUs which were common in at least four of six samples were selected to generate OTUs files. For ileum segments, two SE samples were excluded because of low sequence reads, so ileal OTUs that were common in three of four samples in the group were selected. These newly generated OTU files were used to identify the key OTU communities. NSC analysis was performed on normalized Z-score profiles of OTUs, the amount of shrinkage was determined by cross-validation and test error was minimized. In duodenal NSC analysis, the training error was 0.46 and threshold was 0.43. In ileum NSC analysis, the training error was 0.36 and threshold was 0.63.

### Microbial metabolic function prediction

The PICRUSt (phylogenetic investigation of communities by reconstruction of unobserved states)^33^ was employed to predict functional genes of the classified members of the microbiome (including Cecal OTU data (SRE accession number SRR2537231) obtained from Liu *et al*.^24^) through closed-reference based OTU mapping against the Greengenes database^33^. Mapped closed-reference OTUs are normalized based on the copies of 16S rRNA gene within the known bacterial genomes in Integrated Microbial Genomes (IMG). Predicted genes were clustered hierarchically and categorised on the basis of KEGG^34^ orthologues (KO’s) and pathways (level -3). Using STAMP software^35^. To compare differences in predicted metagenomic functions among different treatment groups, Welch’s *t*-test was applied on the predicted microbiome functions determined by KEGG functional modules (level-3) under various microbiome metabolism^36^.

### Availability of data and supporting materials

The Cecal, duodenal and ileal microbiota OTU sequences have been submitted to Sequence Read Archive (SRA); Cecal OTU sequences with accession number SRR2537231, Duodenal OTU sequences with accession number SRR6312041, and Ileal OTU sequences with accession number SRR6312040.

### Ethics approval

All experiments were performed in accordance with protocols reviewed and approved by the UBC Animal Care Committee (Certificate # A12-0087).

## Results

### Atherosclerotic Lesions on the Intimal Surface of the Aortae

Atherosclerotic lesions of all SUS and RES fed the control diet (SC and RC, respectively) scored 0. Four of six SUS on cholesterol diet (SE) scored 4, the remaining two scored 3+. Three of six RES on the cholesterol diet (RE) scored 0 and the other three scored 1.

### Richness of Small Intestinal Microbiota

#### Duodenum

**Figure 1:**
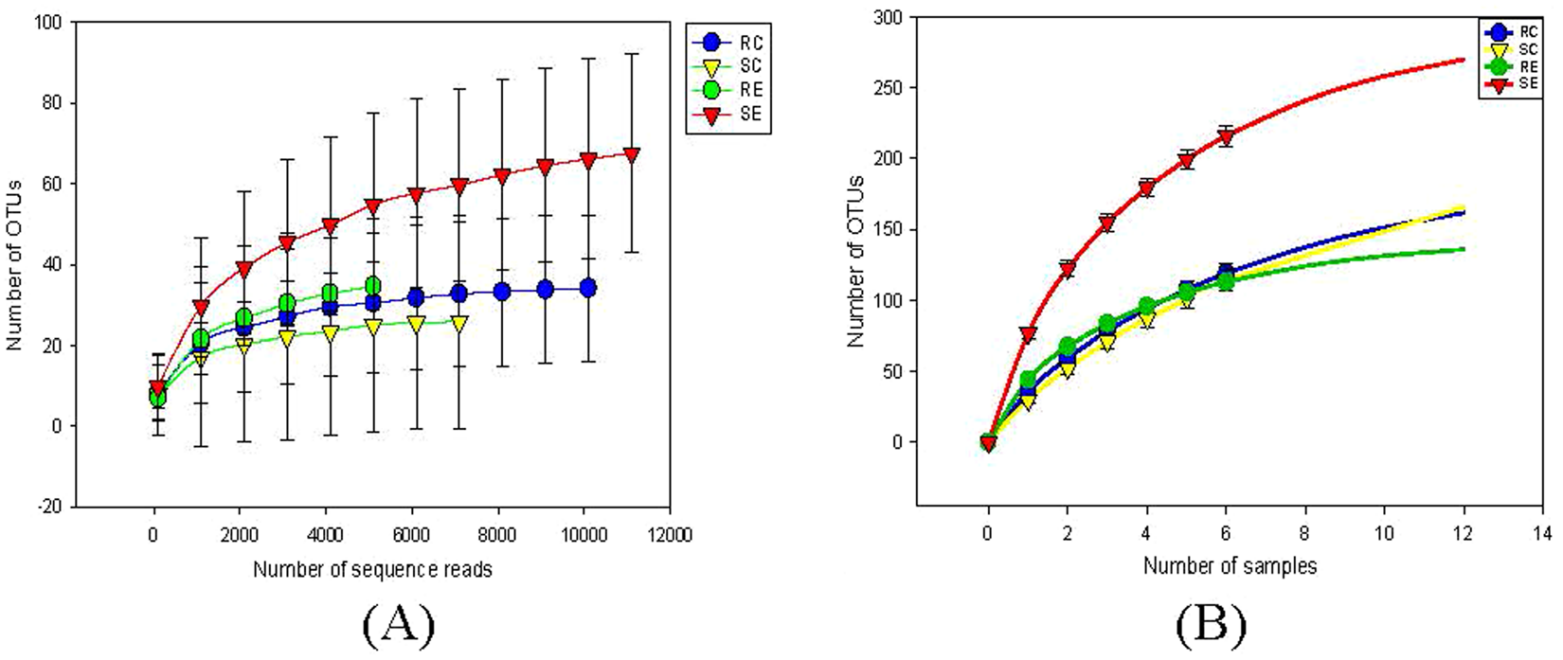
Rarefraction analysis, calculated at 97% dissimilarity, for the assessment of operational taxonomic units (OTU) coverage within the 16S rRNA gene-based duodenal bacterial communities in the RES and SUS quail fed the control or cholesterol diets. (**A**) The number of OTUs as a function of the number of sequence reads. (**B**) The number of OTUs as a function of the number of individual quail sampled.

After quality filteration and trimming, a total of 450,576 sequences were generated with a mean length of 534bp and 18,774 ± 1,633.68 sequences/sample. Rarefaction analysis (Fig. 1) of the sample sizes in the four groups covered most of the OTUs in the sampled population (RC 28.00 ± 4.46; SC 19.40 ± 5.71; RE 44.17 ± 4.78; SE 68.60 ± 8.27). The sequences were classified into 360 species-level operational taxonomic units (OTUs) belong to 15 microbial phyla. Phyla of Firmicutes (95.49%), Proteobacteria (2.67%) and Bacteroidetes (0.83%) dominated in the duodenum where the remaining sequences were identified as the phyla Cyanobacteria, Tenericutes, TM6, Actinobacteria, Chloroflexi, Chlorobi, Acidobacteria, Armatimonadetes, Fusobacteria, Gemmatimonadetes, and Synergistetes, Spirochaetes (together no more than 2% of total sequences).

#### Ileum

**Figure 2:**
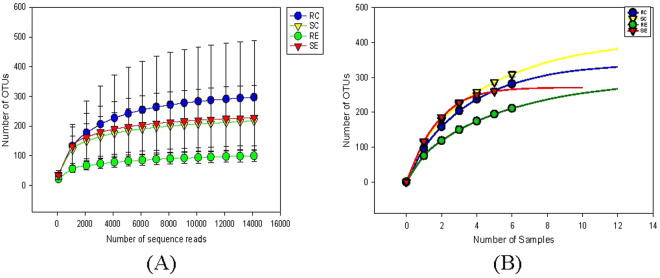
Rarefraction analysis, calculated at 97% dissimilarity, for the assessment of operational taxonomic units (OUT) coverage within the 16S rRNA gene-based ileal bacterial communities in the RES and SUS quail fed the control or cholesterol diets. (**A**) The number of OTUs as a function of the number of sequence reads. (**B**) The number of OTUs as a function of the number of individual quail sampled.

The ileum segment yielded a total of 515,982 sequences with a mean length of 475bp and 23453.73 ± 1104.55 sequences/sample. Rarefaction curve (Fig. 2) reached plateau, showing that the sample size would include most OTUs present in the sampled populations (RC 90.17 ± 19.77; SC 121.50 ± 6.84; RE 105.33 ± 18.18; SE 108.00 ± 30.57). The sequences were classified into 381 OTUs belonging to 8 microbial phyla. The most abundant sequences belonged to four microbial phyla: Firmicutes (73.94%), Proteobacteria (16.28%), Actinobacteria (8.57%) and Bacteroidetes (0.37%). The remaining sequences were identified as Thermi, Acidobacteria, Chlorobi, Chloroflexi, Cyanobacteria, Tenericutes (together less than 2% of total sequences).

### A comparison of Small Intestinal Microbiota Diversity

#### Duodenum

Chao1 species richness was significantly (*P* < 0.05) affected by diet X host genotype interaction. SUS on the cholesterol diet (SE) had the highest OTU richness than the other three treatment groups (SC, RE, and RC) in which no significant difference was detected. There were no significant differences (*P* > 0.05) in Simpson and Shannon estimators among the four treatment groups.

**Figure 3:**
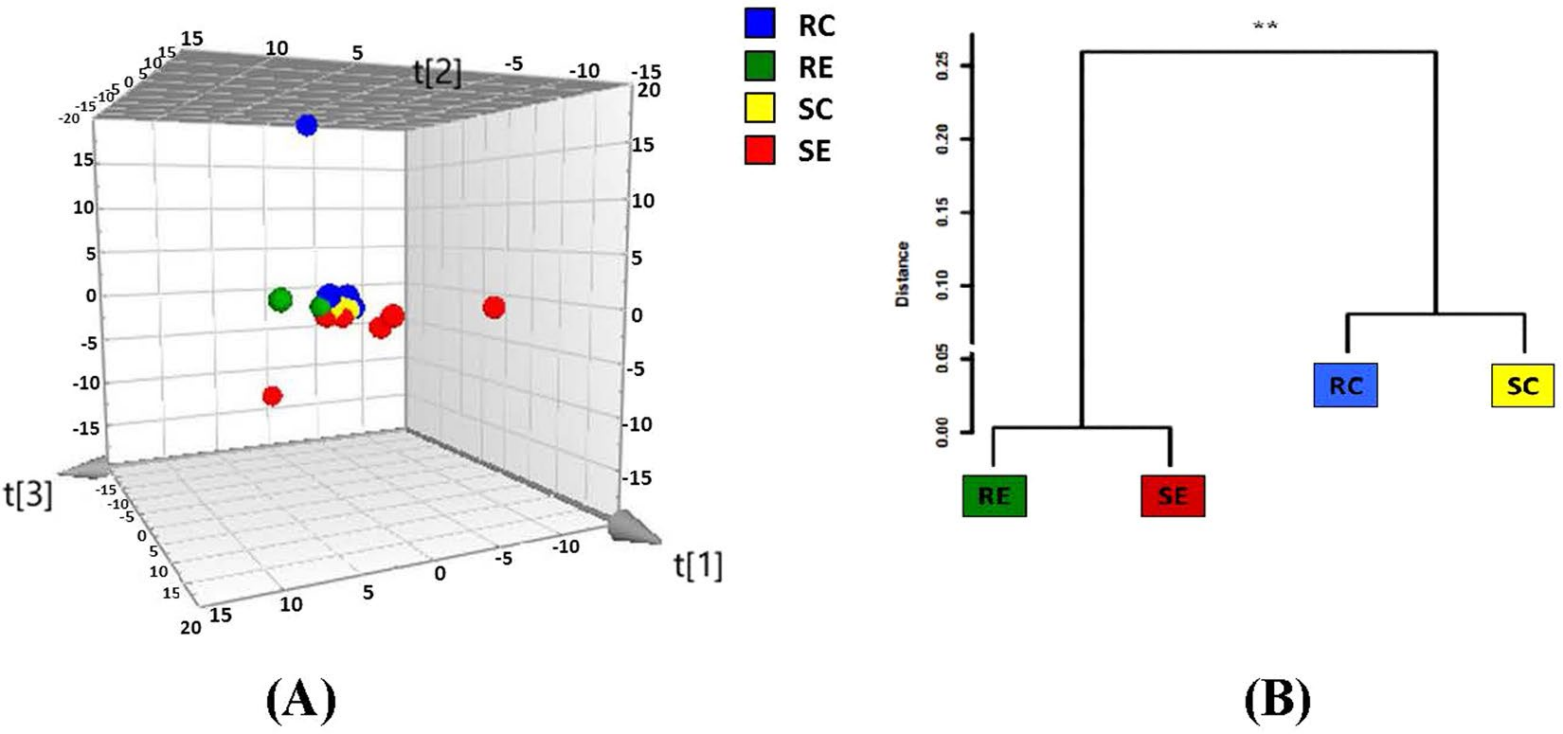
(**A**) Three-dimensional projection of PCA of whole duodenal microbial community. Each symbol represents a single sample. PC^1^ = 0.249; PC^2^ = 0.186; PC^3^ = 0.146 Ellipse: Hotellings’ T2 = 0.97. (**B**) Clustering of duodenal microbiota based on distances between different groups calculated with multivariate analysis of variance test of the first six PCs of the OTU data. The Mahalanobis distances between group means are shown. **P < 0.01.

Although PCA plot of β-diversity showed that the four treatment groups were partly overlapped (Fig. 3A), MANOVA analysis of the duodenal microbial community indicated significant (*P* < 0.01) difference among four treatment groups. A Mahalanobis Distances dendrogram was developed to provide a better visualization of the relationships among the four treatment groups (Fig. 3B). Duodenum samples from birds fed high-cholesterol diet formed a close cluster whereas those from the control diet groups formed another close cluster that was significantly (P < 0.048) distant from the first.

At the genus level, the abundance of *Escherichia* (*Enterobacteriaceae*), *Streptococcus* (*Streptococcaceae*), and *Staphylococcus* (*Staphylococcaceae*) was significantly affected by both diet and host genotype (Table 1). *Escherichia* was most abundant in RC and not different among the other three groups. *Streptococcus* was most abundant in SE but not detectable in SC. *Staphylococcus* was most abundant in SE but not detectable in SC and RE. Unclassified *Enterobacteriaceae* was also found in significant abundance in RC and SE but not detected in SC and RE. There was significantly more unclassified *Rikenellaceae*, unclassified *Coprobacillaceae*, *Blautia*, and *Collinsella* in birds fed the cholesterol diet (SE and RE) than in birds on the control diet (RC and SC). The abundance of *Bacteroides* and *Enterococcus* was significantly affected by host genotype. RES (both RC and RE) had more *Bacteroides* in their duodenum than SUS (SC and SE), while the opposite was true for *Enterococcus* (Table 1).

**Table 1:** Genus level differences in abundance of duodenal microbiota among treatment groups.

	RC	SC	RE	SE	P
*Escherichia* ^§^	4.03 ± 1.05^A^	0.33 ± 0.33^B^	0.63 ± 0.17^B^	0.82 ± 0.17^B^	0.003
*Streptococcus* ^§^	0.64 ± 0.49^AB^	0.00 ± 0.00^B^	0.29 ± 0.21^AB^	1.66 ± 0.60^A^	0.021
*Staphylococcus* ^§^	0.96 ± 0.50^AB^	0.00 ± 0.00^B^	0.00 ± 0.00^B^	2.10 ± 1.15^A^	0.025
Uncl. *Enterobacteriaceae*^§^	0.31 ± 0.19^A^	0.00 ± 0.00^B^	0.00 ± 0.00^B^	0.25 ± 0.16^AB^	0.038
*Alicyclobacillus* ^†^	0.58 ± 0.28	0.00 ± 0.00	0.07 ± 0.07	0.12 ± 0.12	0.059
	**Control diet**		**Cholesterol diet**		**P**
Uncl. Rikenellaceae*	0.00 ± 0.00		0.92 ± 0.36		0.023
Uncl. Coprobacillaceae*	0.51 ± 0.21		3.65 ± 1.49		0.044
Blautia*	0.30 ± 0.16		1.94 ± 0.77		0.045
Collinsella*	0.29 ± 0.21		1.09 ± 0.32		0.050
	**RES**		**SUS**		**P**
*Bacteroides* ^‡^	3.00 ± 0.91		0.20 ± 0.11		0.007
*Enterococcus* ^‡^	0.28 ± 0.17		2.79 ± 1.13		0.047

^§^Significant diet X host genotype interaction.

^†^Effect of diet X host genotype interaction tends to be significant (P < 0.059).

^*^Significant diet effect.

^‡^Significant host genotype effect.

Combining the results of the Venn Diagram/NSC analyses (Table 2), we found that Unclassified *Streptophyta* (ID 4420570) and Unclassified *Lachnospiraceae* (ID 158971) were overabundant in RC but rare in SC. Unclassified *Lactobacillaceae* (ID 292057) was overabundant in RE but rare in SE, while Unclassified *Methylobacterium* (ID 105470) was overabundant in SC but became rare when SUS was fed the cholesterol diet. The abundance of Unclassified *Streptococcaceae* (ID 255359), Unclassified *Lachnospiraceae* (ID 130773), and Unclassified *Sinobacteraceae* (ID 100307) were affected by both the host genotype and dietary cholesterol.

**Table 2:** Summary of key duodenal OTUs characteristics generated by Venn diagram and NSC analysis.

	**RC**	**SC**
**Over-abundant**	Uncl. *Streptococcaceae* (255359) Uncl. *Streptophyta* (4420570) Uncl. *Escherichia* (114510) *Bacteroides fragilis* (3474081) Uncl. *Lachnospiraceae* (158971)**	Uncl. *Enterococcus* (839152) Uncl. *Sinobacteraceae* (100307) Uncl. *Lactobacillus* (137580) Uncl. *Sphingomonas* (4449609) Uncl. *Caulobacteraceae* (4353264) Uncl. *Afipia* (92573)
**Unique**	*Bacteroides ovatus* (2563561) *Pseudomonas stutzeri* (4312845) Uncl. *Ruminococcaceae* (134174) Uncl. *Ruminococcus* (136507)^†^	*Lactobacillus reuteri* (137043)^†^*
	**RE**	**SE**
**Over-Abundant**	Uncl. *Lactobacillaceae* (292057)^‡§^	Uncl. *Lachnospiraceae* (130773) Uncl*. Coprobacillaceae* (592616) Uncl. *Ruminococcaceae* (306633) Uncl. *Ruminococcus* (182245)
**Unique**	Uncl. *RF39* (235065) Uncl. *Sphingomonas* (582921)^†^ Uncl. *Ruminococcaceae* (157546)	Uncl. *Coprobacillaceae* (364722) Uncl. *Ruminococcus* (3438642)^†^ Uncl. *Ruminococcus* (191273)^†^ Uncl. *Ruminococcus* (548503)^†^ Uncl. *Lachnospiraceae* (NCR37)^†^

^‡^Also found as over-abundant in RE ileum.

^†^Unique and over-abundant.

^*^Also found as Unique in RC ceca^24^.

^**^Also found as Abundant in SE ceca^24^.

^§^Also found as Rare in RE ceca and as Abundant in SC ceca^24^.

#### Ileum

**Figure 4:**
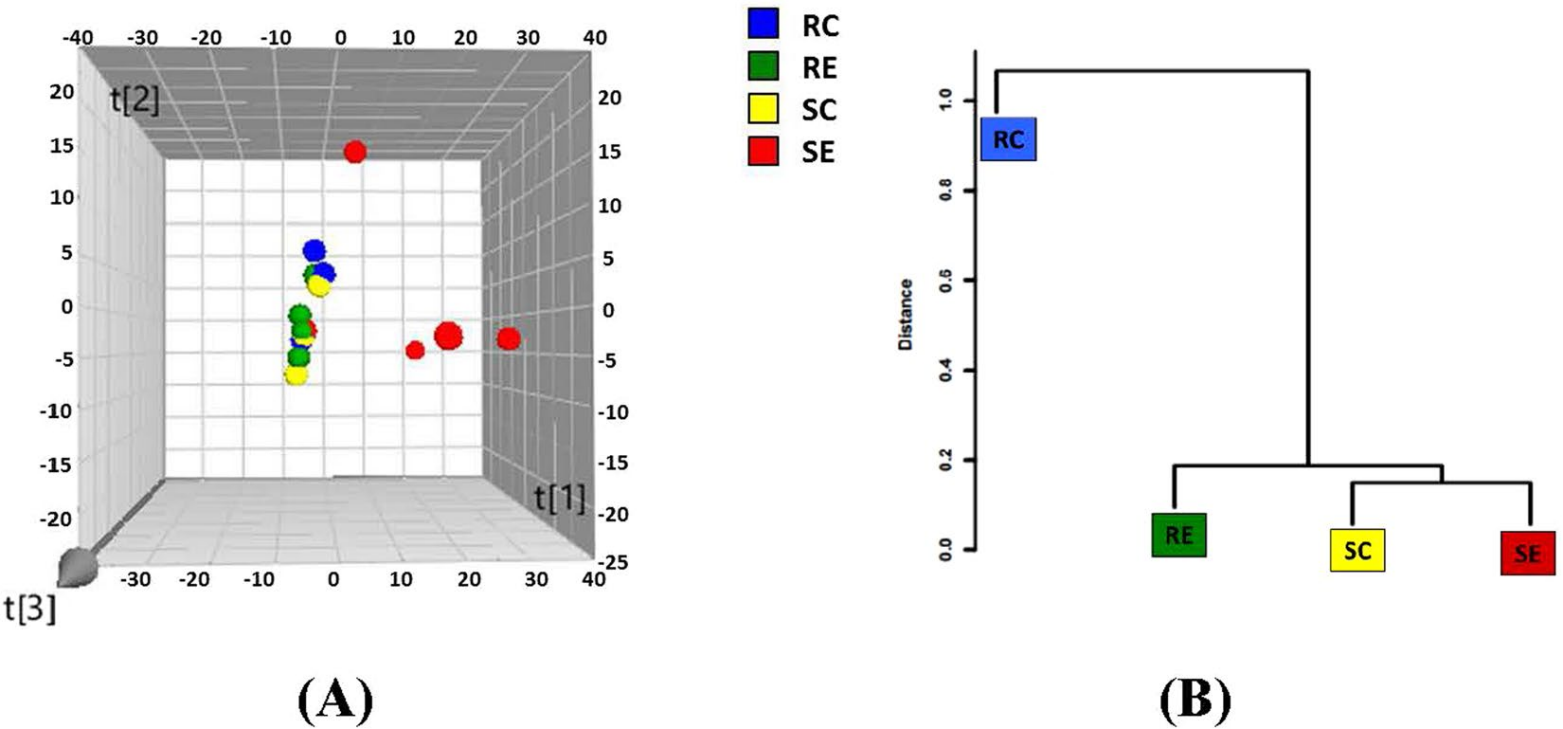
(**A**) Three-dimensional projection of PCA of whole ileal microbial community. Each symbol represents a single sample. PC^1^ = 0.173; PC^2^ = 0.112; PC^3^ = 0.105 Ellipse: Hotellings’ T2 = 0.97. (**B**) Clustering of duodenal microbiota based on distances between different groups calculated with multivariate analysis of variance test of the first six PCs of the OTU data.

There were no significant difference (*P* > 0.05) in α-diversity among the 4 treatment groups according to the Chao1, Simpson and Shannon indices. PCA plot indicated no detectable differences in microbial composition among treatment groups (Fig. 4). MANOVA analysis of variance derived from PCA scores showed there was no statistically significant (*P* > 0.05) separation among the microbiota of the four treatment groups.

At the genus level, only *Lactobacillus* (*Lactobacillaceae*) was significantly (*P* < 0.013) more abundant in RES (51.24 ± 7.01) than in SUS (27.51 ± 2.47).

Summarizing the results of the Venn Diagram/NSC analyses (Table 3), we found that Unclassified *Lactobacillus* (ID 192832) was overabundant in RC but rare in SC. Unclassified *Lactobacillus* (ID 292057) was overabundant in RE but rare in SE. Unclassified *Lactobacillus* (ID 137580) was overabundant in RC but became rare when RES was fed the cholesterol diet.

**Table 3:** Summary of key ileal OTUs characteristics generated by Venn diagram and NSC analysis.

	**RC**	**SC**
**Over-abundant**	Uncl. *Lactobacillus* (137580)* Uncl. *Lactobacillus* (958496) Uncl. *Lactobacillus* (192832) Uncl. *Lactobacillus* (242917)	*Streptococcus alactolyticus* (4473883) Uncl. *Streptococcus* (237444) Uncl. *Acinetobacter* (4482598)
**Unique**	*Lactobacillus reuteri* (354256)	*Propionibacterium acnes* (933896) Uncl. *Hydrogenophilus* (104987) Uncl. *Hydrogenophilus* (575143) Uncl. *Acinetobacter* (4482598) Uncl. *Acinetobacter* (4431922)
	**RE**	**SE**
**Over-abundant**	Uncl. *Lactobacillus* (292057)^‡§^ Uncl. *Lactobacillus* (1142657) Uncl. *Lactobacillus* (823916) Uncl. *Lactobacillus* (574102) Uncl. *Lactobacillus* (4361528) *Acinetobacter guillouiae* (4449456)	
**Unique**	Uncl. *Lachnospiraceae* (158971)** Uncl. *Streptococcus* (15440) Uncl. *Lactobacillus* (332718) Uncl. *Flectobacillus* (539293) Uncl. *Microbacterium* (215095)	*Escherichia coli* (656881) Uncl. *Peptostreptococcaceae* (4409730)^†^ Uncl. *Enterobacteriaceae* (4457268) Uncl. *Enterobacteriaceae* (782953) Uncl. *Enterobacteriaceae* (4454531)

^‡^Also found as Abundant in RE duodenum.

^†^Unique and over-abundant.

^*^Also found as Unique in RC ceca^24^.

^**^Also found as Abundant in SE ceca^24^.

^§^Also found as Rare in RE ceca and as Abundant in SC ceca^24^.

### Association of key bacteria species with plasma lipid parameters

There was a significant diet × genotype interaction in plasma Total Cholesterol (TC) (*P* < 0.0003), LDL (*P* < 0.0002) levels, and LDL/HDL ratio (*P* < 0.0001) (Table 4). SE was significantly higher in these parameters than the other three treatment groups.

**Table 4:** Significant Diet X Host Genotype interaction in plasma lipids level.

Plasma lipids (mmol/L)	N	RES/CON	SUS/CON	RES/CHOL	SUS/CHOL
Total Cholesterol^1^	24	4.49 ± 0.46^a^	5.23 ± 0.31^a^	13.90 ± 1.84^a^	35.84 ± 4.37^b^
Plasma LDL^2^	24	1.04 ± 0.08^a^	1.34 ± 0.08^a^	8.56 ± 2.11^a^	31.39 ± 4.26^b^
Plasma LDL/HDL ratio^3^	24	0.37 ± 0.03^a^	0.42 ± 0.04^a^	2.60 ± 0.74^a^	9.05 ± 1.10^b^

^1^P < 0.0003 Diet X Genotype interaction.

^2^P < 0.0002 Diet X Genotype interaction.

^3^P < 0.0001 Diet X Genotype interaction.

In the duodenum, we have identified 13 bacteria species whose abundance had significant correlation with plasma lipid parameters (Table 5). Nine species have positive correlations with plasma total cholesterol, LDL level or LDL/HDL ratio. Three species showed negative correlation with LDL/HDL ratio and only one species, Unclassified *Lachnospiraceae* (158971), showed positive correlation with HDL. In the ileum, 7 bacteria species have significant correlation with HDL and none showed significant correlation with plasma total cholesterol, LDL level or LDL/HDL ratio (Table 6). Only Unclassified *Lactobacillus* (4414257) showed a negative correlation. None of the bacteria species in the duodenum or ileum had significant correlation with plasma triglycerides level.

**Table 5:** Significant Pearson’s Correlations^§^ between the abundance of duodenum bacteria species and plasma lipid parameters^†^.

**OTUs**	Total Chol	HDL	LDL	LDL/HDL
***Firmicutes***				
Uncl. *Lactobacillus* (NCR39)				0.608*
Uncl. *Ruminococcus* (130103)			0.592*	
*Blautia producta* (158211)	0.609*		0.601*	
Uncl. *Lachnospiraceae* (158971)		0.578*		
Uncl. *Ruminococcaceae* (157804)			0.675*	
Uncl. *Ruminococcaceae* (158217)				0.647*
Uncl. *Ruminococcaceae* (157546)				−0.588*
Uncl. *Ruminococcaceae* (313037)	0.766**		0.744**	0.603*
Uncl. *Collinsella* (NCR69)	0.769**		0.748**	0.620*
Uncl. *Coprobacillaceae* (592616)	0.681*			
***Proteobacteria***				
Uncl. *Afipia* (92573)				−0.653*
Uncl. *Escherichia* (114510)	0.683*		0.693*	0.725**
***Tenericutes***				
Uncl. *RF39* (235065)				−0.609*

^†^RES and SUS fed the cholesterol diet; N = 12.

^§^After elimination of significant correlations due to a single outlier.

*P < 0.05; **P < 0.01.

**Table 6:** Significant Pearson’s Correlations^§^ between the abundance of ileum bacteria species and plasma lipid parameters^†^.

OTUs	HDL
***Firmicutes***	
Uncl. *Lactobacillus* (4414257)	−0.654*
***Proteobacteria***	
Uncl. *Gluconacetobacter* (656881)	0.690*
Uncl. *Acinetobacter* (4361528)	0.748*
Uncl. *Acinetobacter* (1085703)	0.717*
*Acinetobacter guillouiae* (4449456)	0.655*
*Acinetobacter johnsonii* (4333705)	0.782**
Uncl. *Enhydrobacter* (235065)	0.643*

^†^RES and SUS fed the cholesterol diet; N = 10.

^§^After elimination of significant correlations due to a single outlier.

*P < 0.05; **P < 0.01.

### PICRUSt predictions

**Figure 5:**
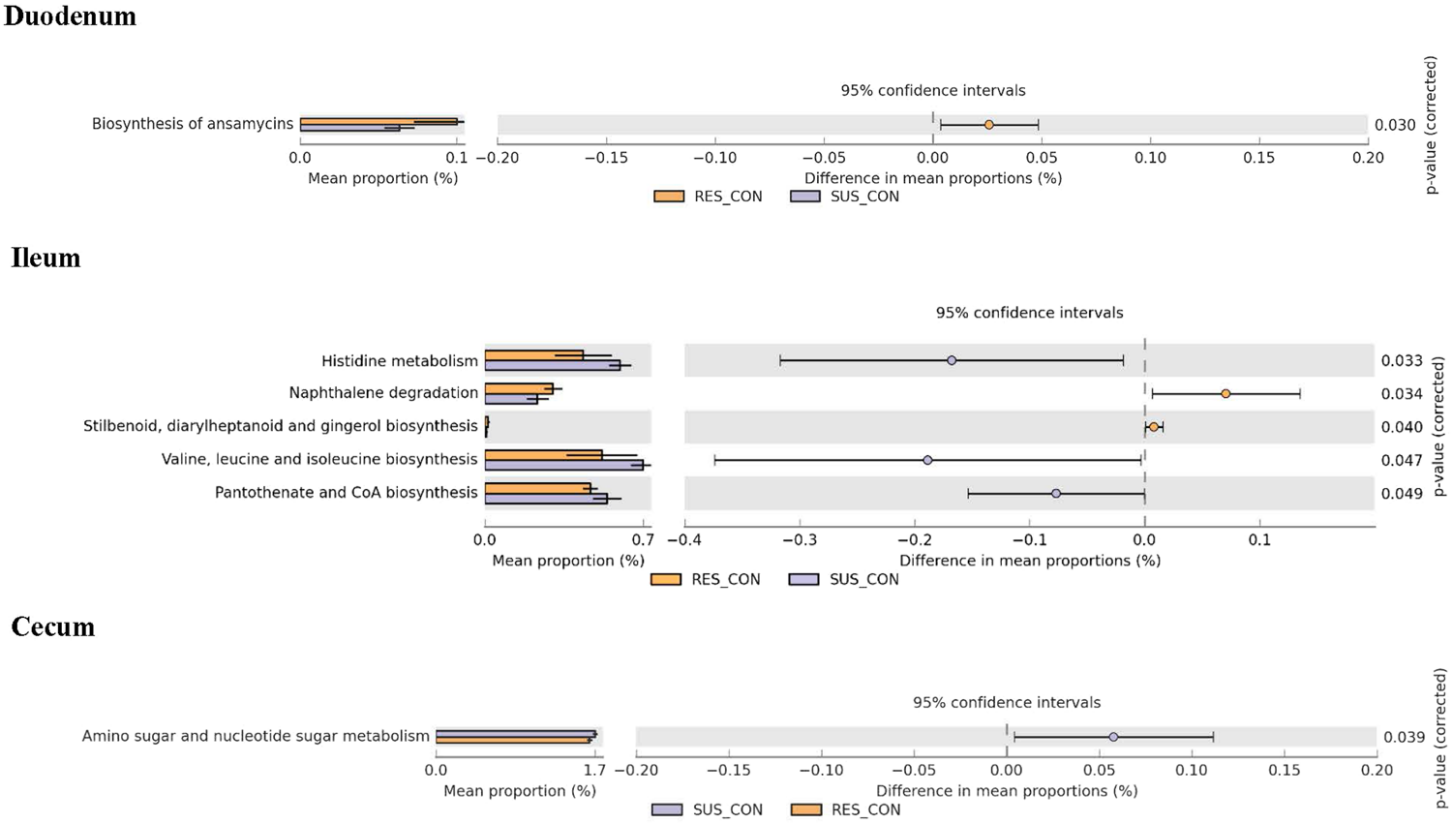
Differences in intestinal microbiome metabolic functions between RC and SC.

When fed the control diet, the RES duodenal microbiome has significantly more functional genes for anasmycinins biosynthesis, and RC has significantly more ileal microbiome genes for naphthalene degradation, stilbenoid, diarylheptanoid and gingerol biosynthesis than SC (Fig. 5). SC, on the other hand, has more Ileal microbiome genes that metabolize Histidine, biosynthesize Valine, leucine, isoleucine, pentothinate and CoA, and a more cecum microbiome genes for amino sugar and nucleotide sugar metabolism.

**Figure 6:**
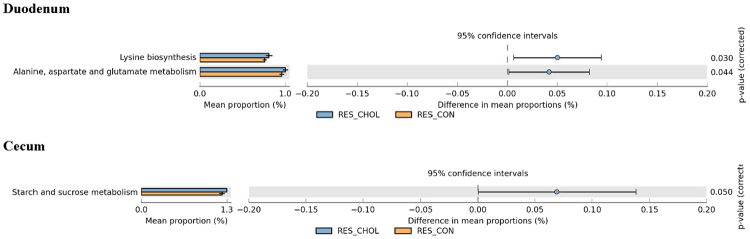
Differences in intestinal microbiome metabolic functions between RC and RE.

**Figure 7:**
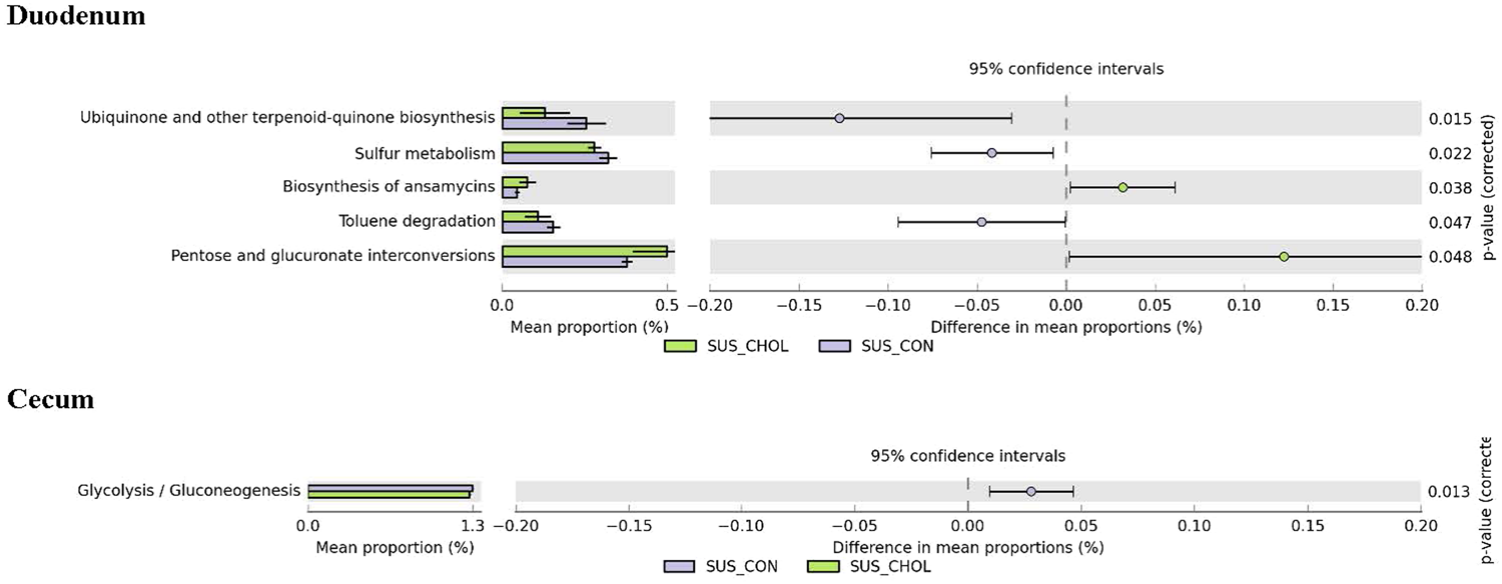
Differences in intestinal microbiome metabolic functions between SC and SE.

In response to the cholesterol diet, there were more duodenal microbiome genes in RE for Lysine biosynthesis, Alanine, aspartate, and glutamate metabolism than in RC (Fig. 6). SUS responded to the cholesterol diet in a different manner (Fig. 7). While there were more duodenal microbiome genes in SE for ansamycines biosynthesis, and pentose and gluconate interconversions than in SC, there were significantly less duodenal microbiome genes in SE for biqinone and other terpenoid-quinone biosynthesis, sulfur metabolism, and toluene degradation than in SC. Note that all these changes in the microbiome genome of the RES and SUS in response to the cholesterol diet were in the duodenum.

**Figure 8:**
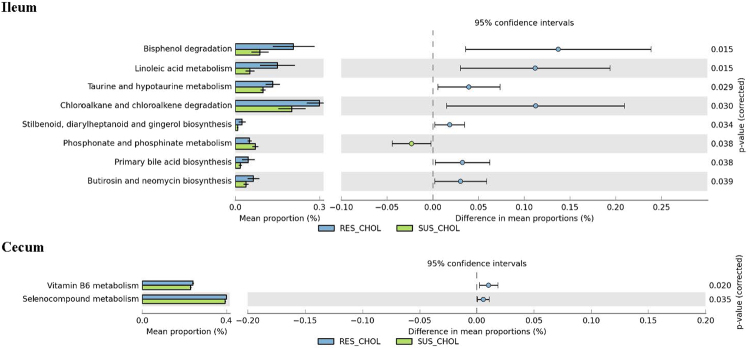
Differences in intestinal microbiome metabolic functions between RE and SE.

When the intestinal microbiome genome of RE and SE were compared, RE had significantly more cecum microbiome genes for Vitamin B6, and selenocompounds metabolism than SE (Fig. 8). RE also had significantly more ileal microbiome genes for bisphenol, chloroalkane and choloroalkene degradation, linoleic acid, taurine and hypotaurine metabolism, Stilbenoid, Diarylheptanoid and gingerol, primary bile acids, butirosin and neomycine biosynthesis compared with SE. On the other hand, SE had significantly more ileum microbiome genes for phosphonate and phosphinate metabolism than RE.

## Discussions

We examined the duodenal and ileal microbiota of 12 week old Japanese quail that had been fed their respective diets for at least 6 weeks. Taxonomic analysis showed that composition of the quail’s duodenal and ileal microbiota at various levels is similar to that of human, mice, hamsters, chickens, emu, and Bobwhite quail^5,37–42^.

### Duodenal microbiota

Although there were 15 microbial phyla found in the quail duodenum, 95% of the sequences detected were Firmicutes. While there was no significant difference in Chao1 richness and Simpson & Shannon diversity between RES and SUS duodenum microbiota when they were fed the regular diet, the cholesterol diet had significantly increased the Chao1 richness of SUS but not the RES quail. The exposure to the cholesterol diet has significantly increased the abundance of Unclassified *Rikenellaceae*, Unclassified *Coprobacillaceae*, *Blautia*, and *Collinsella* in both RES and SUS.

In the human gut, *Collinsella* was enriched in patients with symptomatic atherosclerosis^5^. *Rikenellaceae* are bacteria commensal to the gut and these bacteria thrive on high-fat diets and are enriched in gut microbiomes of obese human. In laboratory mice, a high fat diet also induced a significant increase in *Ruminococcaceae* and *Rikenellaceae*^43^. *Blautia* digest complex carbohydrates. *Blautia* levels were decreased in patients with liver disease and colorectal cancer and children with diabetes. The increased abundance of commensal bacteria belonging to the *Blautia* genus is associated with reduced lethal Graft-versus-Host Disease and improved overall survival^44^.

It is interesting that RES hosted significantly less *Escherichia* and unclassified *Enterobacteriaceae* in their duodenum in response to the cholesterol diet but the SUS was not affected. Many species of *Escherichia* and *Enterobacteriaceae* are known to be pathogenic in human. In mice fed a high-fat diet, there was a significantly higher abundance of *Enterobacteriaceae* and the abundance of *Enterobacteriaceae* correlated with an increase in endotoxin levels in the gut^43^. In chickens, inclusion of probiotic (a mixture of *Bacillus*, *Lactobacillus*, *Streptococcus*, and *Clostridium*) in a high cholesterol diet significantly reduced the number of cecal *Enterobacteriaceae* species^45^. RE hosted an overabundance of Unclassified *Lactobacillaceae* (292057) in the ileum and this may have an effect on combating *Enterobacteriaceae*. Furthermore, RE also uniquely hosted with abundance a species of Unclassified *Sphingomonas* (582921) in the duodenum. Ryu *et al*.^46^ reported isolating many strains of *Sphingomonas sanguis* from wild pheasant GI tracts that could produce antagonistic substance against *Salmonella gallinarum*, the cause of fowl typhoid.

Ability to suppress *Escherichia* and *Enterobacteriaceae* abundance when exposed to a high cholesterol diet may be a defence strategy of RES that was modified in SUS by selective breeding for susceptibility to diet induced atherosclerosis^47–49^. SUS hosted significantly more duodenal *Streptococcus* and *Staphylococcus* in response to the high cholesterol diet whereas RES did not. Many species of *Staphylococcus* are pathogenic in human. A high fat diet will induce the increase of these bacteria in the upper gut^50,51^. We have also demonstrated that the harboring of these bacteria is also affected by the host’s genotype. The difference in defense strategy employed by the two quail strains is also supported by our finding that, regardless of diets, SUS had significantly more *Enterococcus* (pathogenic in human) than RES while RES had significantly more *Bacteroides* than SUS. The inability of SUS to suppress the increase of *Enterobacteriaceae*, *Escherichia*, *and Staphylococcus* may partially facilitate the development of atherosclerosis when exposed to a high cholesterol diet.

### Ileal microbiota

Of the 8 microbial Phyla found in the ileum, 74% were Firmicutes, 16% were Proteobacteria and 8.5% were Actinobacteria. There were no significant difference among the 4 treatment groups in Chao1 richness and Simpson and Shannon diversity. Regardless of diets, RES has significantly more *Lactobacillus* (*Lactobacillaceae*) then SUS. Earlier research on *Lactobacillus* found *L*. *acidophilus* improved egg production, food conversion and reduced the cholesterol concentration in the eggs in chickens^52^. Supplementation with *L*. *rhamnosus* GG decreased serum total cholesterol by 32% in rats with induced hypercholesterolemia^53^. However, the administration of *L*. *fermentum* did not appear to produce a major change in serum lipid fractions in human subjects with elevated serum cholesterol^54^. Recent research has gathered more evidence to show that *Lactobacillus* can be an effective probiotic to lower cholesterol both *in vivo* and *in vitro*^55^. Administration of *L*. *reuteri* NCIMB 30242 significantly reduced serum LDL-C, total cholesterol, and non-HDL cholesterol but not triglycerides and HDL-C in healthy hypercholesterolemic human subjects^56^. It was suggested that *L*. *reuteri* facilitated the deconjugation of intraluminal bile acids to inhibit absorption of non-cholesterol sterols as well as a novel cholesterol-reducing mechanism^56^. It was also demonstrated^57^ that soluble effector molecules produced by *L*. *acidophilus* down regulate the expression of NPC1L, a gene in the small intestine that regulates cholesterol absorption, thus inhibiting the cellular uptake of micellar cholesterol. Fuentes *et al*.^58^ administered a mixture of three strains of *L*. *plantarum* to hypercholesterolemic adults and found significant reduction of plasma total cholesterol and LDL-C. In the ceca, RC also hosted 2 unique species of *Lactobacillus*^24^. *Lactobacillus* species has been commonly used as a probiotic to suppress GI tract inflammation in human^59^. The selection for resistance to diet induced atherosclerosis have also changed the RES gut microbiome when RES is not exposed to dietary cholesterol and may have improved the general gut health of RES^59^.

Our present study found that when exposed to similar diet and microbiological environment, RES was able to harbor significantly more *Lactobacillus* than SUS and thus provided evidence to support the contention that host genotype can affect the gut microbiome^60^.

### Gut Microbiota and Atherosclerosis

A high level of serum total cholesterol is considered to be a risk factor for atherosclerosis. The abundance of Unclassified *Ruminococcaceae* (OTU313037), Unclassified *Collinsella* (NCR69), *Blautia producta* (OTU158211), Unclassified *Escherichia* (OTU114510) in the duodenum is significantly correlated with plasma total cholesterol, LDL levels and LDL/HDL ratio. Only Unclassified *Escherichia* (OTU114510) was found in abundance in the RC duodenum. Liu *et al*.^24^ reported that the abundance of four species of *Lachnospiraceae*, three species of *Ruminococcaceae* and one species of *Coprobacillaceae* in the ceca was positively correlated with plasma Total Cholesterol, plasma LDL, and LDL/HDL ratio. Among these, Unclassified *Ruminococcus* (OTU548503) was found in overabundance in the SE ceca and unique and abundant in the SE duodenum. Another *Ruminococcus* species (OTU191273) was not abundant in the ceca but unique and abundant in the SE duodenum. Interestingly, the abundance of these two species in the ceca was significantly correlated with the plasma lipids level but not significantly so when they were in the duodenum. None of the microbiota species in the ileum was significantly correlated with TC, LDL, or LDL/HDL. The abundance of 5 *Proteobacteria* species in the ileum was significantly correlated with plasma HDL level but none of these species were found in over-abundance in the ileum. Further research on the functionality of these bacteria with relationship to their abundance and the intestinal environment is warranted^61–63^ to better our understanding of gut microbial activity.

RES has higher abundance of *Lactobacillus* in the ileum than SUS. The administration of *L*. *acidophilus* ATCC 4356 was found to protect against atherosclerosis in ApoE knock-out mice through the inhibition of intestinal cholesterol absorption^64^. The administration of *L*. *plantarum* 299v to smokers also resulted in the reduction of several cardiovascular disease risk factors^65^.

In comparison with SE, RE has significantly more microbiome genes for Vitamin B6 metabolism in their ceca. Pyridoxal 5′ phosphate (PLP), the active form of vitamin B6, is involved in a wide variety of physiologic processes including gluconeogenesis and the synthesis of sphingolipids and neurotransmitters. It also functions as a cofactor for many enzymes required for amino acid metabolism^66–68^. Complex sphingolipids are essential structural components of intestinal membranes, providing protection and integrity to the intestinal mucosa and regulating intestinal absorption processes. The role of sphingolipid signaling has been established in numerous cellular events, including intestinal cell survival, growth, differentiation, and apoptosis^68,69^. An important biochemical change associated with vitamin B6 deficiency is that of hyperhomocysteinemia which was recognized as a cardiovascular event risk factor^67^. The oxidative stress due to low level of vitamin B6 accelerates the development of homocysteine-induced atherosclerosis in rats^70^.

RE had significantly more microbiome genes than SE in the ceca that are involved with selenocompound metabolism. Selenomethionine is the major selenocompound in cereal grains, grassland legumes and soybeans. Methylseleninic acid (MASIV) has been proposed to be a nutritional selenium source^71^. Nutritional selenocompounds are considered to be transformed into the common intermediate selenide for utilization as selenoenzymes. Selenium affects expression of 15% of the genes that participate in lipid metabolism, especially in lipid transport, including apolipoprotein. It is a major determinant of the capacity of HDL to promote cholesterol efflux. Plasma HDL levels are inversely correlated with atherosclerosis^72^.

In the ileum, the RE microbiome is significantly better in bisphenol degradation than the SE microbiome. Bisphenol A (BPA) has been reported to cause hypercholesterolemia. BPA caused an overexpression of key genes (*Mvd*, *Lss*, *Hmgcr*, and *Sqle*) involved in cholesterol biosynthesis. BPA also induced the expression of the sterol regulatory element-binding proteins 2, a master regulator of hepatic cholesterol biosynthesis^73^.

Another microbiome metabolic function that was significantly better in the RE ileum than the SE ileum was Linoleic acid metabolism. Intestinal bacteria can metabolize linoleic acid to form vaccenic acid or 10-hydroxy-18:1. Both of these compounds are precursors of conjugated linoleic acid (CLA, also known as Omega-6)^74^. CLA improves blood lipids by lowering triglycerides and cholesterol levels. Deficiency in dietary Linoleic acids increased the risk of Coronary Heart Disease and obesity^75,76^.

Another two related microbiome metabolic functions that were performed significantly better in the RE ileum than in the SE ileum were primary bile acid biosynthesis, and taurine and hypotaurine metabolism. The synthesis of bile acids is the major pathway of cholesterol catabolism in mammals and most vertebrate. Although several of the enzymes involved in bile acid synthesis are active in many different cell types, the liver is the only organ in mammals where complete biosynthesis of primary bile acids (cholic acid, chenodeoxycholic acid) can occur. Secondary bile acids (e.g. lithocholic acid, deoxycholic acid), on the other hand, can be biosynthesized by intestinal microbiota. The prediction by PICRUSt that the ileal microbiota in quail can biosynthesize primary bile acids seems to contradict current knowledge. However, Kim *et al*.^77^ reported biosynthesis of primary bile acids in a variety of marine bacterial taxa. Therefore, it may be that excessive dietary cholesterol can stimulate some ileal microbiota to completely or incompletely biosynthesize primary bile acids. Bile acids have long been known to facilitate digestion and absorption of lipids in the small intestine as well as regulate cholesterol homeostasis^78^. It has now become clear that bile acids are also hormones involved in the regulation of various metabolic processes. Through activation of various signaling pathways, bile acids regulate not only their own synthesis and enterohepatic circulation, but also triglyceride, cholesterol, glucose, and energy homeostasis^79,80^.

Taurine is a major constituent of bile and can be found in the large intestine. It has many fundamental biological roles, such as conjugation of bile acids, antioxidation, osmoregulation, membrane stabilization, and modulation of calcium signaling. It is essential for cardiovascular function, and development and function of skeletal muscle, the retina, and the central nervous system. Taurine has been shown to reduce the secretion of apolipoprotein B100 and lipids in HepG2 cells. High concentrations of serum lipids and apolipoprotein B100 (essential structural component of VLDL and LDL) are major risk factors of atherosclerosis and coronary heart disease^81^.

Finally, the RE ileal microbiome was significantly better than the SE ileal microbiome in butirosin and neomycin biosynthesis. Both butirosin and neomycin are antibiotics. Neomycin is not adsorbed through the gastrointestinal wall and has been used as a preventive measure for hypercholesterolemia in human^82^.

Thus it seems that microbiome in the ileum and ceca of RES contributed significantly towards the resistance to diet induced atherosclerosis.

Excessive dietary cholesterol has significantly affected the duodenal microbiome of SUS. In comparison with SC, duodenal microbiome of SE had significantly less Ubiquinone and other terpenoid-quinone biosynthesis. Ubiquinone, or Coenzyme Q10, shares a biosynthetic pathway with cholesterol^83^. In human, CoQ_10_ deficiency may be associated with a multitude of diseases including heart failure^84^. The severity of heart failure correlates with the severity of CoQ_10_ deficiency. Emerging data suggest that the harmful effects of reactive oxygen species are increased in patients with heart failure and CoQ_10_ may help to reduce these toxic effects because of its antioxidant activity. However, only a few randomized controlled trials have compared CoQ_10_ to other therapeutic modalities, and no systematic review of existing randomized trials has been conducted. There was “no conclusive evidence to support or refute” the use of ubiquinone for the treatment of heart failure^84^. A 2009 Cochrane review concluded that studies looking at the effects of CoQ10 on blood pressure were unreliable, and therefore no conclusions could be made regarding its effectiveness in lowering blood pressure. A couple of Terpenoid-Quinones isolated from plants have found to possess significant antihyperglycemic activity^85^.

The duodenal microbiome of SE also had significantly less sulfur metabolism ability. Sulfur is generally acquired in the diet through protein^86^. Gut microbes require sulfur inputs and, because of their active metabolism and tremendous number, are likely to play a major role in the metabolism of luminal sulfur. Sulfur is either converted to sulfated compounds, assimilated by host cells or excreted. The importance of the microbiota and the metabolism of sulfated bile acids are now established, but further work is needed to understand how dietary fat intake influences these pathways. In addition, one essential and another conditionally essential amino acid (methionine and cysteine, respectively) are sulfated. Cysteine and methionine are used by the body to make glutathione (GSH). GSH is an important antioxidant, preventing damage to important cellular components caused by reactive oxygen species. Hoekstra *et al*.^87^ examined cultured aortic endothelial cells from RES and SUS and found that SUS cells were more susceptible to oxidative stress than RES cells. In particular, SUS cells had significantly lower level of GSH.

Thus, in terms of metabolic functions, the selective breeding for susceptibility to diet induced atherosclerosis has mainly affected the microbiome in the duodenum while selection for resistance has mainly affected the microbiome in the ileum and ceca.

### Gut microbiome of RES and SUS on control diet

RES and SUS were developed by divergent selective breeding and the selection criteria were based on the atherosclerotic plaque score of males fed a high cholesterol diet^21^. Males with the highest plaque scores were selected as breeders to establish the SUS strain and males with the lowest scores were selected to be breeders to establish the RES strain. After this first generation, selection was done within strain and there was no crossing back to the foundation population. SE males in the SUS strain with the highest plaque scores were selected as breeders to propagate the next generation of SUS. RE males in RES strain with the lowest plaque scores were used for breeding in the RES. The change in gut microbiome in the males fed a high cholesterol diet is considered to be a correlated response as selection was not done on the microbes. The change in gut microbiome in males not exposed to a high cholesterol diet is also a correlated trait as selection was not done on birds fed the regular diet.

In our study, RC duodenal microbiome was predicted to be significantly better than the SC duodenal microbiome in the biosynthesis of ansamycins. Ansamycin antibiotics are a class of microbial metabolites that exhibit an array of biological activities. It has been postulated that Ansamycins can be used to control microbes that are involved in systemic inflammatory processes^88^. Ansamycins are highly effective against *Chlamydia pneumoniae* which plays important role in the occurrence and development of coronary heart disease^89^. The microbiome in the RC ileum also has more genes than SC for naphthalene degradation. Naphthalene has serious deleterious effects on bird health^90,91^. Strains of *Pseudomonas putida* and *Escherichia coli* carry the genes for naphthalene degradation on a recombinant plasmid pRKJ3^92,93^. In general, gut microbiome of RES (both RE and RC) facilitated better health of their host more so than their SUS counterparts.

The ileal microbiome of SC, on the other hand, has significantly more genes for histidine metabolism, valine/leucine/isoleucine biosynthesis, and pantothenate/CoA biosynthesis than RC. Many gut bacteria, especially those in *Clostridia* and *Fuscobacteria*, have been identified to metabolize histidine^94^. Histidine can be metabolized through 5 different pathways which give rise to several important metabolic products such as histamine and formiminoglutamic acid (FIGLU). The N^10^-formyl tetrahydrofolic acid (THF) is essential in protein synthesis in microorganisms^94,95^. Two-component signal transduction systems are the major routes bacteria use to detect environmental signals that mediate changes in cellular behavior or biological processes. These systems consist typically of two proteins—a sensory histidine kinase and a response regulator^96^. Pantothenic acid (Vitamin B5) is an essential vitamin required for the biosynthesis of Coenzyme A (CoA) and CoA functions as a carrier of acyl groups in enzymatic reactions involved in synthesis of fatty acids, cholesterol, and sterols. CoA also has an essential function in lipid metabolism^97,98^. The gene clusters involved in amino acid biosynthetic pathways have been described in *lactobacillus*, and systems controlling gene expression have been identified^96^. Leucine, isoleucine and valine are branched-chain amino acids (BCAA). There is an increasing body of evidence that the functional output of the gut microbiota, in particular bacterial metabolites like amino acids, are important modulators of host physiology^97^. BCAA leucine, isoleucine and valine are preferred substrates of gut bacteria to generate a complex mixture of metabolic end products such as short-chain fatty acids (SCFA). Some of these have been shown to modulate bacterial gene expression for amino acid metabolism and also to modulating the mucosal immune system of the host^98,99^. The most abundant SCFA are acetate, propionate and butyrate. Once absorbed, butyrate is used by liver cells for gluconeogenesis and cholesterol synthesis^100^. Furthermore, it has been postulated that chronic elevation of systemic BCAA levels, as seen in obesity, impair transport of these BCAA from the intestine lumen into systemic circulation.

Thus leading to more amino acid catabolism in the lumen and more SCFA formation. Thus, it seems that selection for susceptibility to atherosclerosis in SUS has made them more efficient in the absorption and synthesis of fatty acids, cholesterol, and sterols.

## Conclusions

Our study has well controlled independent variables to provide conclusive evidence on the interaction of host genotype and diet on gut microbiota. The SUS and RES quail strains are a result of divergent genetic selection from a common foundation population^21^. The eggs of the four treatment groups were artificially incubated at the same time in close proximity in the same incubator. Birds of the two strains fed the same diet were raised in the same pen. Blood and tissue sampling was done at the same age for all the birds^24^. The difference in gut microbiota in the two quail strains fed the same diet can therefore be attributed to host genotype. The gut microbiota difference in birds of the same strain fed different diets can be attributed to dietary effect. The difference in the diets is only in the level of cholesterol and so the dietary effect can be attributed to the effect of cholesterol. Difference among the 4 treatment groups can be attributed to the interaction between host genotype and diet.

The interaction between host genotype and diet provided possible explanations for the varying efficacy of probiotic treatments^101,102^. Of particular interest is the overabundance of *Lactobacillus* in the ileum of RES. With the help of PICRUSt predictions, we can relate gut microbiota with the host’s resistance and susceptibility to diet induced atherosclerosis. Atherosclerosis is a complex disease affected by the interaction of genetic and environmental risk factors. To understand better the role that gut microbiota play in the development of atherosclerosis, it will be worthwhile to examine the gene expression of the intestinal wall and the liver in association with these microbiome genes.

## Electronic supplementary material

Supplementary Information
