# Supplementary material for: Interaction of genotype and diet on small intestine microbiota of Japanese quail fed a cholesterol enriched diet
Source: Sci Rep. 2018 Feb 5;8:2381. doi: 10.1038/s41598-018-20508-9 (PMC5799165; doi:10.1038/s41598-018-20508-9)
Supplement: Supplementary file 1 — Supplementary Information [file 41598_2018_20508_MOESM1_ESM.doc]

**Interaction of genotype and diet on small intestine microbiota of Japanese quail fed a cholesterol enriched diet**

Shasha Liu^1, 2§^, Hein Min Tun^3, 4§^, Frederick C. Leung^3^, Darin C. Bennett^2, 5^,

Hongfu Zhang^1‡^ and Kimberly M. Cheng^2*^

^1^The State Key Laboratory of Animal Nutrition, Institute of Animal Sciences, Chinese Academy of Agricultural Sciences, Beijing, China. ^2^Avian Research Centre, Faculty of Land and Food Systems, University of British Columbia, Canada. ^3^School of Biological Sciences, Faculty of Science, University of Hong Kong, Hong Kong SAR, China. ^4^Department of Pediatrics, University of Alberta, Canada. ^5^Animal Science Department, California Polytechnic State University, San Luis Obispo, California, U.S.A.

Supplementary Information: **Unique duodenal OTUs generated by Venn diagram**

|  | **ID** | **Phylum** | **Class** | **Order** | **Family** | **Genus** | **Species** |
| --- | --- | --- | --- | --- | --- | --- | --- |
| **RC** |  |  |  |  |  |  |  |
|  | 1110791 | *Bacteroidetes* | *Sphingobacteriia* | *Sphingobacteriales* | *Chitinophagaceae* | unclassified |  |
|  | 2563561 | *Bacteroidetes* | *Bacteroidia* | *Bacteroidales* | *Bacteroidaceae* | *Bacteroides* | *ovatus* |
|  | 68458 | *Proteobacteria* | *Alphaproteobacteria* | *Rhizobiales* | *Methylobacteriaceae* | *Methylobacterium* | *reuteri* |
|  | 541859 | *Proteobacteria* | *Gammaproteobacteria* | *Pseudomonadales* | *Pseudomonadaceae* | *Pseudomonas* | uncl. |
|  | 4312845 | *Proteobacteria* | *Gammaproteobacteria* | *Pseudomonadales* | *Pseudomonadaceae* | *Pseudomonas* | *stutzeri* |
|  | 3385021 | *Firmicutes* | *Bacilli* | *Bacillales* | *Staphylococcaceae* | *Staphylococcus* | uncl. |
|  | 573035 | *Firmicutes* | *Bacilli* | *Bacillales* | *Alicyclobacillaceae* | *Alicyclobacillus* | uncl. |
|  | 134174 | *Firmicutes* | *Clostridia* | *Clostridiales* | *Ruminococcaceae* | unclassified |  |
|  | 583089 | *Firmicutes* | *Clostridia* | *Clostridiales* | *Lachnospiraceae* | *Blautia* | uncl. |
|  | 136507 | *Firmicutes* | *Clostridia* | *Clostridiales* | *Lachnospiraceae* | *Ruminococcus* | uncl. |
| **SC** |  |  |  |  |  |  |  |
|  | 141350 | *Firmicutes* | *Bacilli* | *Lactobacillales* | *Enterococcaceae* | unclassified |  |
|  | 137043 | *Firmicutes* | *Bacilli* | *Lactobacillales* | *Lactobacillaceae* | *Lactobacillus* | *reuteri* |
|  | 284413 | *Proteobacteria* | *Gammaproteobacteria* | *Xanthomonadales* | *Sinobacteraceae* | unclassified |  |
| **RE** |  |  |  |  |  |  |  |
|  | 582921 | *Proteobacteria* | *Alphaproteobacteria* | *Sphingomonadales* | *Sphingomonadaceae* | *Sphingomonas* | uncl. |
|  | 782953 | *Proteobacteria* | *Gammaproteobacteria* | *Enterobacteriales* | *Enterobacteriaceae* | *Escherichia* | uncl. |
|  | 4449609 | *Proteobacteria* | *Alphaproteobacteria* | *Sphingomonadales* | *Sphingomonadaceae* | *Sphingomonas* | uncl. |
|  | NCR108 | *Proteobacteria* | *Gammaproteobacteria* | *Xanthomonadales* | *Sinobacteraceae* | unclassified |  |
|  | 157546 | *Firmicutes* | *Clostridia* | *Clostridiales* | *Ruminococcaceae* | unclassified |  |
|  | 235065 | *Tenericutes* | *Mollicutes* | *RF39* | unclassified |  |  |
| **SE** |  |  |  |  |  |  |  |
|  | 211212 | *Firmicutes* | *Clostridia* | *Clostridiales* | *Lachnospiraceae* | unclassified |  |
|  | NCR91 | *Firmicutes* | *Bacilli* | *Lactobacillales* | *Streptococcaceae* | *Streptococcus* | *alactolyticus* |
|  | 3244896 | *Firmicutes* | *Bacilli* | *Lactobacillales* | *Streptococcaceae* | *Streptococcus* | *luteciae* |
|  | 287813 | *Firmicutes* | *Bacilli* | *Lactobacillales* | *Streptococcaceae* | unclassified |  |
|  | NCR13 | *Firmicutes* | *Bacilli* | *Lactobacillales* | *Lactobacillaceae* | *Lactobacillus* | uncl. |
|  | NCR39 | *Firmicutes* | *Bacilli* | *Lactobacillales* | *Lactobacillaceae* | *Lactobacillus* | uncl. |
|  | NCR50 | *Firmicutes* | *Bacilli* | *Lactobacillales* | *Lactobacillaceae* | *Lactobacillus* | *salivarius* |
|  | NCR98 | *Firmicutes* | *Bacilli* | *Lactobacillales* | *Lactobacillaceae* | *Lactobacillus* | *salivarius* |
|  | NCR118 | *Firmicutes* | *Bacilli* | *Lactobacillales* | *Lactobacillaceae* | unclassified |  |
|  | 3438642 | *Firmicutes* | *Clostridia* | *Clostridiales* | *Lachnospiraceae* | *Ruminococcus* | uncl. |
|  | 191273 | *Firmicutes* | *Clostridia* | *Clostridiales* | *Lachnospiraceae* | *Ruminococcus* | uncl. |
|  | NCR37 | *Firmicutes* | *Clostridia* | *Clostridiales* | *Lachnospiraceae* | unclassified |  |
|  | 166637 | *Firmicutes* | *Clostridia* | *Clostridiales* | *Lachnospiraceae* | *Ruminococcus* | uncl. |
|  | 548503 | *Firmicutes* | *Clostridia* | *Clostridiales* | *Lachnospiraceae* | *Ruminococcus* | uncl. |
|  | 158217 | *Firmicutes* | *Clostridia* | *Clostridiales* | *Ruminococcaceae* | unclassified |  |
|  | 364722 | *Firmicutes* | *Erysipelotrichi* | *Erysipelotrichales* | *[Coprobacillaceae]* | unclassified |  |
|  | 229069 | *Firmicutes* | *Erysipelotrichi* | *Erysipelotrichales* | *Erysipelotrichaceae* | *Eubacterium* | *dolichum* |
|  | 107044 | *Bacteroidetes* | *Bacteroidia* | *Bacteroidales* | *Rikenellaceae* | unclassified |  |
|  |  |  |  |  |  |  |  |
